# The Impact of Sarcopenia on Glucose and Insulin Dynamics: A Mathematical Model

**DOI:** 10.2174/0115733998419601251007101814

**Published:** 2025-10-24

**Authors:** Abdesslam Boutayeb, Mohamed Lamlili E.N, Wiam Boutayeb

**Affiliations:** 1 Department of Mathematics, Emirates Aviation University, Dubai, UAE;; 2 Department of Mathematics, Faculty of Sciences, Mohamed I University, Oujda, Morocco;; 3 Department of Mathematics**,** Higher School of Education and Training, Mohamed I University, Oujda, Morocco

**Keywords:** Diabetes, sarcopenia, physical activity, mathematical modelling, glucose, insulin sensitivity

## Abstract

**Introduction:**

Diabetes and sarcopenia are major age-related health issues in the world. Due to their bidirectional relationship, they significantly impact the quality of life of elderly men and women, especially those over 60 years old. In this brief communication, we incorporate the effect of sarcopenia into a previously developed mathematical model examining the impact of exercise on the dynamics of glucose and insulin.

**Methods:**

Extending a previous mathematical model by Derouich and Boutayeb, derived from the minimal model of Bergman, we present a new mathematical model using ordinary differential equations in which the parameter “S” is included to express the additive effect of sarcopenia.

**Results:**

Considering that the sarcopenia effect is introduced multiplicatively, the new model proposed shows that sarcopenia lowers insulin sensitivity both in the presence and absence of physical exercise. The effects of sarcopenia on glucose dynamics are illustrated by simulations with varying model parameters.

**Discussion:**

A large number of clinical studies have shown the link between Type 2 diabetes and sarcopenia. Some authors have particularly explored the causal relationship between insulin resistance and sarcopenia. The proposed mathematical model indicates that sarcopenia reduces insulin sensitivity in both the presence and absence of physical exercise, and that severe sarcopenia may negate the benefits of physical exercise.

**Conclusion:**

By theoretically demonstrating the relationship between sarcopenia and Type 2 diabetes, this mathematical model confirms the interrelationship between sarcopenia and Type 2 diabetes for any population worldwide, regardless of its regional, cultural, or environmental specificity.

## 1 INTRODUCTION

Type 2 diabetes mellitus (T2DM) and sarcopenia constitute serious age-related disorders affecting developed and developing countries and exacerbating the disease burden, especially in low- and middle-income countries [1].

According to the recent data released by the International Diabetes Federation (IDF), the global number of adults aged 20-79 years living with diabetes reached 589 million in 2024 [2]. Besides the diabetes-related health expenditure, which is more than one trillion USD, the burden of diabetes also includes some 3.5 million annual deaths and large numbers of complications, including kidney failure, eye diseases, amputations, and cardiovascular diseases in general. The IDF Atlas 11^th^ edition estimated that the worldwide comparative diabetes prevalence was 9.8% with a high regional disparity varying from 5.3% in the African region to 11.9% in Middle-East and North Africa (MENA) where six countries (Pakistan, Kuwait, Qatar, Saudi Arabia, Egypt and Bahrain) appear among the top 10 countries with age-standardised diabetes prevalence in adults (20-79 years) in 2024 [2].

Following the definition of sarcopenia published in 2010, the European Working Group on Sarcopenia in Older People 2 (EWGSOP2) updated this definition in 2018 through three criteria: (1) low muscle strength, (2) low muscle quantity or quality, and (3) low physical performance. The EWGSOP2 suggested the following three stages of sarcopenia: (a) probable sarcopenia (criterion 1), (b) confirmed diagnosis (by additional documentation of criterion 2), and (c) severe sarcopenia (criteria 1, 2, and 3) [3]. However, despite being a serious disease affecting large numbers of women and men worldwide, there is no agreement on a single diagnostic criterion of sarcopenia and, consequently, the few studies dealing with the global prevalence of sarcopenia show high levels of heterogeneity. Indeed, a systematic review published in 2021 by Petermann-Rocha Fanny *et al.* compared the results yielded by eligible studies, including 263 narrative syntheses, of which 151 were meta-analyses, and found a prevalence of sarcopenia ranging from 10% to 27% among the selected studies. The prevalence varied between 8% and 36% in individuals <60 years, while it ranged from 10% to 27% in individuals≥60 years [4].

While studies have clinically shown a correlation between sarcopenia and different diseases, such as diabetes, kidney diseases, heart failure, and cerebrovascular diseases [5], many experts also suggest that sarcopenia may cause unpredictable health outcomes like falls, fractures, frailty, reduced quality of life, increased morbidities, and mortality [3, 6].

An abundant literature is devoted to mathematical modelling for diabetes, as indicated by a multitude of regular reviews [7-11]. Mathematical modelling for sarcopenia attracts many publications [12-15]. However, to the best of our knowledge, there is no mathematical model that simultaneously deals with diabetes and sarcopenia. Hence, the current short communication represents the first attempt at a project aimed at developing different mathematical models that address the bidirectional relationship between diabetes and sarcopenia.

## 2 METHODS

In this short communication, we modify a previous mathematical model published by Derouich and Boutayeb in 2002 on “The effect of physical exercise on the dynamics of glucose and insulin” by introducing a new parameter *S* between 0 and 1, standing for different levels of sarcopenia and varying from the highest level of sarcopenia (*S* close to 0) to the lowest level (*S* close to 1).

The mathematical model published by Derouich and Boutayeb was governed by the following differential Eq. (**1**) [16]:


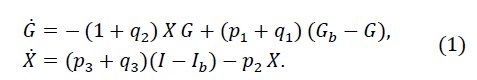


Here *G*(*t*) stands for the glucose concentration, *I*(*t*) and *X*(*t*) represent the plasma insulin and interstitial insulin; *p_1_*, *p_2_* and *p_3_* are parameters defined by Bergman *et al.* [17], while the parameters related to physical activity (*q_1_*, *q_2_* and *q_3_*) were defined by Derouich and Boutayeb as follows: *q_1_*: the effect of physical exercise in accelerating the utilization of glucose by muscles and the liver, *q_2_*: the effect of physical exercise in increasing the muscular and liver sensitivity to the action of insulin, and *q_3_*: the effect of physical exercise on increasing the utilization of insulin.

### 2.1 The new mathematical model

Based on large clinical studies suggesting that sarcopenia simultaneously lowers muscle mass, insulin-signalling capacity, and oxidative disposal, and given that these limitations act in parallel on every insulin-dependent pathway [18], we modify the first equation of Model 1 by scaling the uptake term (*1 + q_2_)XG* to obtain the new mathematical Model 2, as given in Eq. (**2**):


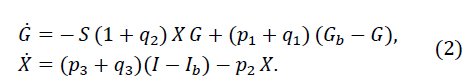


The parameter *S* ϵ [0, 1] indicates the effect of sarcopenia on the dynamics of glucose and insulin. For *S* = 1, Model 2 reduces to Model 1, while for *S* < 1, every insulin-driven glucose sink is proportionally weakened, consistent with empirical data showing that post-prandial disposal is approximately linearly proportional to lean mass.

## 3 RESULTS

### 3.1 Effect of Sarcopenia on Insulin Sensitivity

In line with the association between sarcopenia and insulin resistance, our model indicates that sarcopenia reduces insulin sensitivity. Recapitulating and extending the results given by Derouich and Boutayeb [16], for insulin sensitivity, we obtain the following four expressions, as given in Eqs. (**3**) and (**4**):


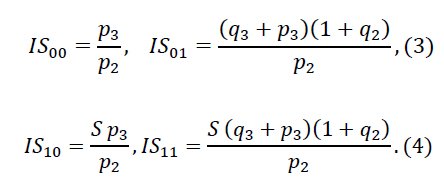


Since all the parameters are non-negative and *S* ≤ 1, a straightforward comparison shows that:

- *IS_10_ < IS_00_*: In the absence of physical exercise, sarcopenia lowers insulin sensitivity.
- *IS_00_ < IS_01_*: In the absence of sarcopenia, physical exercise improves insulin sensitivity.
- *IS_11_ < IS_01_*: Sarcopenia lowers insulin sensitivity in the presence of physical exercise.
- *IS_11_ < IS_00_*: Severe sarcopenia negates the effect of physical exercise on insulin sensitivity.

### 3.2 Simulation

The parameters used for simulating glucose dynamics under sarcopenia and exercise are listed in Table **1**. They are adapted from the original minimal model [17], and its exercise-enhanced extension [16], with different values used for simulation. The system of ordinary differential equations was solved in Python (v3.11) using the solve_ivp routine from the SciPy library with the Runge-Kutta method of order 5(4) (RK45), and tolerances set to rtol = 1×10^−8^ and atol = 1×10^-10^.

In Fig. (**1**), we compare glucose trajectories between individuals with normal muscle mass (*S*= 1.0) and severe sarcopenia (*S* = 0.4), under the same exercise intensities. We observe that the curve of the healthy, inactive person and that of the sarcopenic individual under strong exercise are nearly superimposed, suggesting that the benefits of physical activity are cancelled by severe sarcopenia.

Tables (**2**-**4**) present the glucose levels at 60 and 120 minutes, as well as the insulin sensitivity index *IS*, for different sarcopenia levels and exercise intensities.

## 4 DISCUSSION

As indicated in the introduction section, a large number of clinical studies have shown the link between sarcopenia and T2DM. More precisely, many authors have explored a causal relationship between insulin resistance and sarcopenia [18, 19], a bidirectional relationship between type 2 diabetes mellitus (T2DM) and sarcopenia [20], a mutual interrelationship between T2DM and sarcopenia [1], and sarcopenia as both a cause and consequence of metabolic dysregulation [21, 22].

Our mathematical model shows clearly how sarcopenia is linked to diabetes through action on the dynamics of glucose and insulin. It indicates that sarcopenia lowers insulin sensitivity both in the presence and absence of physical exercise, and that severe sarcopenia may cancel the benefits of physical exercise.

Our simulations demonstrate the impact of sarcopenia on glucose and insulin dynamics in three cases (Fig. **1**): (1) no or low physical activity, (2) moderate exercise, and (3) high level of physical exercise. In the three cases (although at different levels), sarcopenia appears as a serious cause of type 2 diabetes and its common complications.

Consequently, our model constitutes a theoretical and pragmatic tool susceptible to help health decision makers to elaborate efficient and cost-effective strategies aiming to help and convince especially men and women aged 60 years or over to avoid sarcopenia as far as possible by adopting healthy lifestyle behaviours, notably by adhering to a high-quality balanced diet and a regular physical activity adapted to age and gender. This suggestion is all the more plausible given the lack of intervention strategies and programs dealing with sarcopenia.

## CONCLUSION AND PERSPECTIVES

The simple mathematical model presented in this short communication explains at least one way in which sarcopenia relates to diabetes, confirming the results reported in the clinical literature and highlighting the interrelationship between sarcopenia and T2DM for any population worldwide, regardless of its regional, cultural, or environmental specificity. For elderly people, maintaining muscle mass and strength can be achieved through regular physical activities, such as walking. Interestingly, walking is also the most affordable form of exercise for elderly people living with diabetes. The model proposed in this short communication is expected to be improved by other models that incorporate more differential equations and include S as a variable S(t). Compartimental models are also under consideration to analyse and predict the evolution of individuals between compartments, such as healthy people (H), sarcopenic people (S), diabetics without complications (D), and diabetics with complications (C).

## AUTHORS’ CONTRIBUTIONS

The authors confirm their contributions to the paper as follows: A.B.: contributed to the research design, draft writing, and reference checking. M.L.E.N.: contributed to the research design, simulations, figure plotting, and text editing. W.B.: contributed to the research design, data and parameters checking, and draft revision. All authors reviewed the results and approved the final version of the manuscript.

## Figures and Tables

**Fig. (1):**
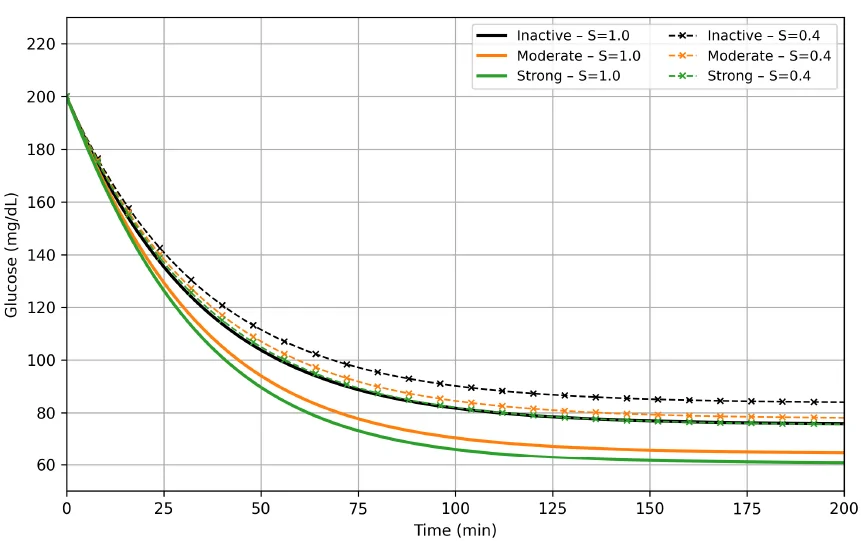
Glucose trajectories for healthy individuals (*S* = 1.0) and individuals with severe sarcopenia (*S* = 0.4), under three exercise intensities.

**Table 1:** Simulation parameters and values used in the sarcopenia-exercise model.

**Parameter**	**Value**	**Units**	**Comment**
*p_1_*	0.028	min^-1^	Glucose effectiveness
*p_2_*	0.025	min^-1^	Insulin decay rate
*p_3_*	1.2 × 10^-5^	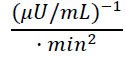	Insulin action gain
*G_b_*	90	mg/dL	Basal glucose
*I_b_*	12	μU/mL	Basal insulin
*G_0 _*	220	mg/dL	Initial post-load glucose
*X_0 _*	0.002	min^-1^	Initial insulin effect
*I*	20	μU/mL	Constant insulin input
*q_1_* (No or lower)	2×10^-6^	-	Glucose gain
*q_2_* (No or lower)	0.20	-	Multiplicative uptake
*q_3_* (No or lower)	2×10^-6^	-	Insulin sensitivity gain
*q_1_* (Moderate)	1×10^-5^	-	Glucose gain
*q_2_* (Moderate)	0.65	-	Multiplicative uptake
*q_3_* (Moderate)	9×10^-6^	-	Insulin sensitivity gain
*q_1_* (Strong)	3×10^-5^	-	Glucose gain
*q_2_* (Strong)	0.95	-	Multiplicative uptake
*q_3_* (Strong)	1×10^-5^	-	Insulin sensitivity gain

**Table 2:** Glucose levels and insulin sensitivity for no or lower exercise intensity.

*S*	**Glycemia after 60 min [mg/dL]**	**Glycemia after 120 min [mg/dL]**	**Insulin Sensitivity [10^-4^ min^-1^]**
1.0	96.4	78.9	7.0
0.8	99.0	81.5	5.6
0.6	101.7	84.3	4.2
0.4	104.5	87.3	2.8
0.2	107.5	90.5	1.4

**Table 3:** Glucose levels and insulin sensitivity for moderate exercise intensity.

*S*	**Glycemia after 60 min [mg/dL]**	**Glycemia after 120 min [mg/dL]**	**Insulin Sensitivity [10^-4^ min^-1^]**
1.0	85.9	67.7	14.0
0.8	90.2	71.8	11.2
0.6	94.7	76.3	8.4
0.4	99.6	81.5	5.6
0.2	104.9	87.3	2.8

**Table 4:** Glucose levels and insulin sensitivity for strong exercise intensity.

*S*	**Glycemia after 60 min [mg/dL]**	**Glycemia after 120 min [mg/dL]**	**Insulin sensitivity [10^-4^ min^-1^]**
1.0	81.4	63.4	17.0
0.8	86.3	67.9	13.6
0.6	91.6	73.1	10.2
0.4	97.4	79.0	6.8
0.2	103.6	85.8	3.4

## Data Availability

The data and supportive information are available within the article.
